# The Promise of Multicancer Early Detection. Comment on Pons-Belda et al. Can Circulating Tumor DNA Support a Successful Screening Test for Early Cancer Detection? The Grail Paradigm. *Diagnostics* 2021, *11*, 2171

**DOI:** 10.3390/diagnostics12051243

**Published:** 2022-05-17

**Authors:** Eric A. Klein, Tomasz M. Beer, Michael Seiden

**Affiliations:** 1 Glickman Urological and Kidney Institute, Cleveland Clinic Lerner College of Medicine, 9500 Euclid Ave, Q10-1, Cleveland, OH 44195, USA; 2 Knight Cancer Institute, Oregon Health & Science University, CH-14R, 3303 SW Bond Ave, Portland, OR 97239, USA; beert@ohsu.edu; 3 Independent Researchers, Phialdelphia, PA 19111, USA; mvseiden@gmail.com

**Keywords:** multicancer early detection, methylation, circulating cell-free genome atlas

## Abstract

Multicancer Early Detection (MCED) represents a new and exciting paradigm for the early detection of cancer, which is the leading cause of death worldwide. Current screening tests, recommended for only five cancer types (breast, lung, colon, cervical, and prostate), are limited by a lack of complete adherence to guideline-based use and by the fact that they have cumulative high false positive rates. MCED tests agnostically detect cancer signals in the blood with good sensitivity and low false positive rates, can predict the cancer site of origin with high accuracy, can detect highly lethal cancers that have no current screening tests, and promise to improve cancer screening by improving efficiency and reducing the overall number needed to screen. Herein we outline this promise and clarify several published misconceptions about this field.

## 1. Introduction

Multicancer Early Detection (MCED) represents a new and exciting paradigm for the early detection of cancer. We read with interest Pons-Belda et al.,’s [1] commentary on the potential role of ctDNA as a test for early cancer detection. We share the authors’ enthusiasm for this technology and wish to clarify some of the points raised in their article.

## 2. Understanding Tumor Biology in the Context of MCED

**Figure 1:**
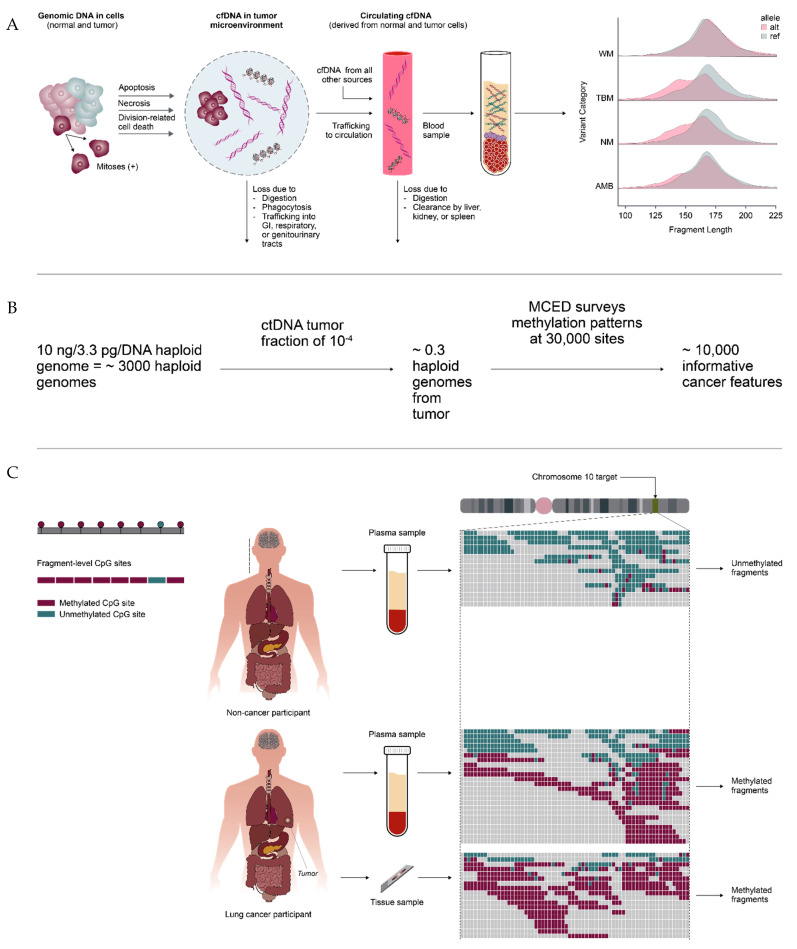
(**A**) cfDNA is composed of fragmented DNA, not individual genomes. (**B**) MCED surveys many sites enabling detection at or below a ctDNA tumor fraction of 10^−4^ from one blood tube. (**C**) Each MCED classifier feature covers multiple bases, typically with reinforcing methylation patterns across multiple CpGs.

The most important issue to be discussed is the lower limit of detectability of ctDNA assays and whether this can be defined by mutant allele fraction (MAF) estimated from tumor size. While the most important property and limiting factor for sensitivity of liquid biopsies is the number of copies of tumor origin available in a sample for detection, it is a fundamental error to use MAF alone as suggested by Pons-Belda to estimate cancer target abundance because it ignores the reality that cfDNA is highly fragmented. The authors err by assuming that cfDNA is composed of contiguous whole genomes, when in fact individual haploid genomes generate millions of ∼160 bp fragments, each of which may be sampled by the assay (Figure 1A). To assess the potential for an MCED to detect cancer at low (10^−4^) tumor fraction, both the number of informative fragments and the noise level of the fragments must be considered. The design of GRAIL’s commercial test, Galleri, exploits cfDNA fragmentation by determining methylation patterns targeting 30,000 independently informative regions. Each region considers methylation patterns across multiple differentially methylated CpGs. Galleri’s ability to detect tumor fractions below 10^−4^ arises from this broad coverage of multiple targets, conferring both low noise levels and high specificity (Figure 1B,C). In fact, it is notable that no published MCED looks at only one site in the genome to determine if a cancer signal is present.

Beyond the consideration of fragmentation, while it may be true in general that smaller tumors shed less DNA, there are differences in detectable cfDNA levels that are determined by factors independent of tumor size. In a multivariate analysis of CCGA participants [2] that included mitotic and metabolic activity, grade, and lymph node status, the results show for breast and lung cancer that only tumor mitotic volume and metabolic activity, and not tumor size, predicted for MAF. For colorectal cancer, the surface area of tumors invading beyond the subserosa, and not tumor size, was the only factor that predicted for MAF. We have also shown that while MAF increases with stage across cancer types, it varies by orders of magnitude within a given type and stage [3]. Significant differences in MAF are also observed among cancer types, with high-mortality cancers (esophageal, gastric, hepatobiliary, lung, and pancreatic) having higher MAF than low-mortality cancers within each stage. Finally, tumors not detected by Galleri have an improved prognosis over tumors that are detected across all stages [4].

In Table 1 of the Pons-Belda commentary, the authors extrapolate MAF from nonsmall cell lung cancer (their reference 36) and a theoretic article (their reference 37) that highlights that the number of cells in each tumor volume varies across tumors because of “…deformation and variability of extracellular spaces modify the density of cell packings. Furthermore, any tumor contains, in variable proportions, macrophages, lymphoid cells, etc. Thus, any tabulation of definite tumor cell numbers per unit of tumor tissue volume is likely to be misleading” [5]. They then go on, erroneously in our view, to compare these estimates to the sensitivity of mammography, which of course detects a completely different tumor type and has a cumulative 10-year false positive rate as high as 51% [6]. Certainly no real conclusions can be drawn from such a spurious comparison.

It is apparent from these multiple observations that estimates of MAF based on tumor size alone are insufficient to understand the complex biology of cfDNA, and as such relying on such estimates leads to false conclusions of MCED performance.

## 3. Other Issues

**Figure 2:**
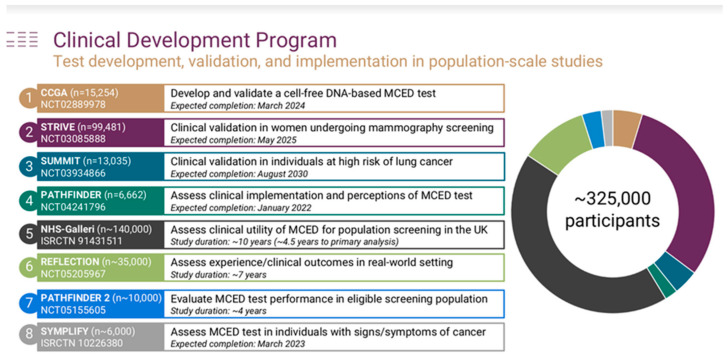
Clinical Development Program of a cfDNA-based Multicancer Early Detection (MCED) Test.

Beyond these issues, there are several additional misunderstandings in the commentary by Pons-Belda et al. that we would also like to address. First, Galleri is based on whole genome methylation using a targeted assay that covers the most informative regions of the genome for cancer detection and cancer signal origin prediction [7]. This approach was chosen because it had the best performance for both cancer detection and cancer site of origin (CSO) prediction after a head-to-head comparison to whole genome sequencing and targeted mutation assays [8]. CSO prediction for the top two sites of cancer ranged from 88–92% [7,9]. The authors have also significantly understated GRAIL’s commitment to clinical evaluation of this MCED test, which includes a global effort across eight studies totaling more than 300,000 participants using a variety of study designs (Figure 2).

## 4. Conclusions

The current regimen of USPSTF-recommended screening tests have been adopted because of their ability to reduce cancer mortality. Despite their widespread adoption, there are still approximately 600,000 cancer-related deaths per year in the US alone [10]. MCED tests promise to mitigate many of the shortfalls of the current screening paradigm which include:

- A total of 71% of all cancers are not found because of a lack of an established screening test [11];
- Unscreened cancers account for ~70% of cancer-related deaths [11];
- Patients are more likely to be diagnosed with a different cancer than the one targeted by screening [12];
- Adherence rates are suboptimum (5–80%) [13];
- Positive predictive value for single cancers is <10% [11];
- Cumulative false positive rates are very high (40–50%) [14,15].

A simple blood test that detects multiple cancer types (especially those that currently lack any effective screening) is likely to improve both access and adherence and reduce the death rate. Modeling studies, for example, have estimated that when used as an adjunct to current screening tests, Galleri has the potential to avert more than a quarter of these deaths [11]. MCED tests represent the future of cancer screening.

## References

[1] Pons-Belda O.D., Fernandez-Uriarte A., Diamandis E.P. (2021). Can Circulating Tumor DNA Support a Successful Screening Test for Early Cancer Detection? The Grail Paradigm. Diagnostics.

[2] Bredno J., Lipson J., Venn O., Aravanis A.M., Jamshidi A. (2021). Clinical correlates of circulating cell-free DNA tumor fraction. PLoS ONE.

[3] Jamshidi A., Liu M., Klein E.A., Venn O., Hubbell E., Beausang J.F., Zhang N., Kurtzman K.N., Hou C., Richards D.A. (2021). Evaluation of Cell-Free DNA (cfDNA) Multi-Omics Approaches for Multi-Cancer Early Detection. Ann. Oncol..

[4] Chen X., Dong Z., Hubbell E., Kurtzman K.N., Oxnard G.R., Venn O., Melton C., Clarke C.A., Shaknovich R., Ma T. (2021). Prognostic Significance of Blood-Based Multi-cancer Detection in Plasma Cell-Free DNA. Clin. Cancer Res..

[5] Del Monte U. (2009). Does the cell number 10_9_ still really fit one gram of tumor tissue?. Cell Cycle.

[6] Myers E.R., Moorman P.G., Gierisch J.M., Havrilesky L.J., Grimm L., Ghate S.V., Davidson B., Mongtomery R.C., Crowley M.J., McCrory D.C. (2015). Benefits and Harms of Breast Cancer Screening. JAMA.

[7] Klein E., Richards D., Cohn A., Tummala M., Lapham R., Cosgrove D., Chung G., Clement J., Gao J., Hunkapiller N. (2021). Clinical validation of a targeted methylation-based multi-cancer early detection test using an independent validation set. Ann. Oncol..

[8] Liu M.C., Oxnard G.R., Klein E.A., Swanton C., Seiden M.V., CCGA Consortium (2020). Sensitive and specific multi-cancer detection and localization using methylation signatures in cell-free DNA. Ann. Oncol..

[9] Nadauld L., McDonnell C., Beer T., Liu M., Klein E., Hudnut A., Whittington R., Taylor B., Oxnard G., Lipson J. (2021). The PATHFINDER Study: Assessment of the Implementation of an Investigational Multi-Cancer Early Detection Test into Clinical Practice. Cancers.

[10] Siegel R.L., Miller K.D., Fuchs H.E., Jemal A. (2022). Cancer statistics, 2022. CA Cancer J. Clin..

[11] Hubbell E., Clarke C.A., Aravanis A.M., Berg C.D. (2021). Modeled Reductions in Late-stage Cancer with a Multi-Cancer Early Detection Test. Cancer Epidemiol. Biomark. Prev..

[12] Clarke C.A., Hubbell E., Ofman J.J. (2021). Multi-cancer early detection: A new paradigm for reducing cancer-specific and all-cause mortality. Cancer Cell.

[13] Smith R.A., Oeffinger K.C. (2020). The Importance of Cancer Screening. Med. Clin. N. Am..

[14] De Koning H.J., Van Der Aalst C.M., De Jong P.A., Scholten E.T., Nackaerts K., Heuvelmans M.A., Lammers J.-W.J., Weenink C., Yousaf-Khan U., Horeweg N. (2020). Reduced Lung-Cancer Mortality with Volume CT Screening in a Randomized Trial. N. Engl. J. Med..

[15] (2019). National Lung Screening Trial Research Team Lung Cancer Incidence and Mortality with Extended Follow-up in the National Lung Screening Trial. J. Thorac. Oncol..

